# Conservative Treatment of Interstitial Ectopic Pregnancy with the Combination of Mifepristone and Methotrexate: Our Experience and Review of the Literature

**DOI:** 10.1155/2020/8703496

**Published:** 2020-08-01

**Authors:** Guglielmo Stabile, Federico Romano, Francesca Buonomo, Giulia Zinicola, Giuseppe Ricci

**Affiliations:** ^1^Institute for Maternal and Child Health IRCCS “Burlo Garofolo”, Trieste, Italy; ^2^Department of Medicine, Surgery and Health Sciences, University of Trieste, Trieste, Italy

## Abstract

**Introduction:**

Interstitial pregnancy (IP) is an ectopic pregnancy (EP) located in the portion of the fallopian tube that penetrates the uterine muscular layer. Incidence increased in the last two decades with the widespread use of the assisted reproductive techniques. It is estimated in 1-6% of all the EPs, with a maternal mortality rate of 2.0-2.5%. Clinical presentation, gestational age at diagnosis, beta-human chorionic gonadotropin (*β*-hCG) levels, ultrasound features, and patient preference, should be considered to determine the best management: surgical, medical treatment, or close observation. We report two cases of IP successfully managed with systemic MTX and Mifepristone: in one case *β*-hCG was >10.000 mIU/mL and a vital embryo was present.

**Materials and Methods:**

A literature search was carried out on MEDLINE, EMBASE, and PUBMED. We identified two cases of IP referred to the Institute for Maternal and Child Burlo Garofolo, Trieste. Data related to clinical presentation, *β*-hCG, and ultrasound scan at the moment of the diagnosis were recorded. In one of the cases, the *β*-hCG level was >10.000 mIU/mL, and a vital embryo was testified at an ultrasound scan. The patient was asymptomatic and she was treated using multidose systemic Methotrexate (MTX) combined with Mifepristone. In the second case, in the presence of a clinically stable patient with *β* − hCG > 10.000 mIU/mL, it was chosen that the administration of Mifepristone combined with a double dose of MTX. *β*-hCG levels and ultrasound examinations were performed weekly until a complete resolution of the IP.

**Results:**

In the first case, *β*-hCG dropped down in 5 days and became undetachable in 30 days. In the second case, *β*-hCG became undetectable in 47 days. The first-line therapy in asymptomatic women could be addressed to a combined protocol, consisting of a systemic multidose MTX regimen with a single oral dose of Mifepristone.

**Conclusions:**

Clinical management of IP remains a debated topic. In selected cases, a systemic multidose MTX regimen combined with a single oral dose of Mifepristone could be considered also in the presence of high serum *β*-hCG.

## 1. Introduction

Interstitial pregnancy (IP) is an unusual form of ectopic pregnancy (EP) consisting of a gestational sac (GS) that implants in the interstitial portion of the fallopian tube as it passes through the myometrium. Its incidence is estimated in 1-6% of all the EPs [1]. IP represents approximately 2–4% of all the tubal pregnancies, occurring in 1/2,500–5,000 of live births [2], with a maternal mortality rate of 2-2.5% [3]. The most common risk factor for IP is assisted reproduction techniques, followed by tubal and uterine anomalies, which can be induced by endometriosis and uterine leiomyoma, a prior salpingectomy, a previous EP, and a history of pelvic inflammatory disease. During the conventional laparoscopic salpingectomy, aseptic inflammation associated with electrocoagulation may cause embryonic migration and implantation into the uterine horn. Multiple factors should be considered to determine which are the best management for this issue: medical treatment, close observation, or surgical treatment by laparoscopy or laparotomy. These factors include clinical presentation, gestational age at diagnosis, *β*-hCG serum level, contraindications to medical therapy, and patient preference. Medical treatment is considered a good alternative especially when fertility needs to be preserved. Nowadays, it is still unclear which is the best medical approach to IP. We report two cases of IPs successfully managed with systemic MTX and Mifepristone. To our knowledge, this is the first case of IP with *β* − hCG > 10.000 mIU/mL and a vital embryo treated with this therapeutic scheme.

## 2. Materials and Methods

A literature search was carried out in March 2020 using the keywords “interstitial pregnancy”, “medical treatment”, “methotrexate”, and “mifepristone”. Articles that were published from January 1991 until December 2019 were obtained from MEDLINE, EMBASE, and PUBMED.

We present 2 cases of interstitial pregnancy in hemodynamically stable women at an early gestational age successfully treated with medical therapy using Methotrexate and Mifepristone.

**Case 1 .**

**Figure 1:**
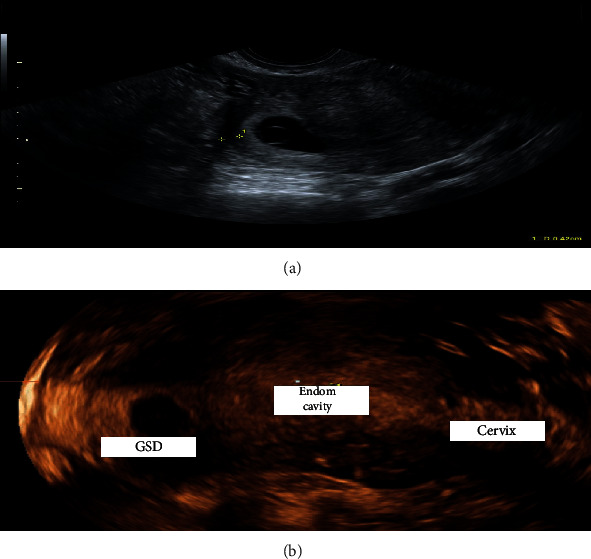
(a) Myometrium thickness; (b) reconstruction 3D: GSD: gestational sac; Endom cavity: endometrial cavity.

A 32-year-old Caucasian pregnant nulliparous woman with a history of two previous miscarriages treated with dilatation and curettage was referred to our center, Institute for Maternal and Childbirth Burlo Garofolo, with recent slight vaginal bleeding. Based on her last menstrual period, she was 7 weeks pregnant. The transvaginal sonography (TVUS) revealed the possible diagnosis of an IP with a GS located eccentrically close to the right uterine horn, and an embryo with a crown-rump length (CRL) of 3.6 mm with cardiac activity. A 3-D-reconstruction confirmed our diagnosis based on a myometrium layer around the GS of 4 mm and an empty uterine cavity (Figure 1). Her serum *β*-hCG was 19,397 mIU/mL, so we arranged for immediate hospitalization. We discussed all possible approaches with the patient, considering, on one side, the high *β*-hCG serum levels and on the other the fact that she was utterly asymptomatic. We opted for a medical strategy, which combined 600 mg of oral mifepristone with a multidose systemic MTX regimen [3], consisting of an intramuscular (IM) injection of 1 mg/kg of body weight every two days, balanced with 0.1 mg/kg of folinic acid, activated on the days of MTX injections. After 2 days, *β*-hCG raised to 21,716 mIU/mL, while the patient remained hemodynamically stable and \TVUS did not show any difference. The day after the second injections of MTX, she referred moderate metrorrhagia, *β*-hCG declined to 16,000 mIU/mL and the TVUS confirmed the interruption of the pregnancy. Five days later, *β*-hCG dropped down to 1,264 mIU/mL and became entirely negative in one month. Ultrasound examinations were performed weekly until a complete resolution of IP (Table 1).

**Case 2 .**

**Figure 2:**
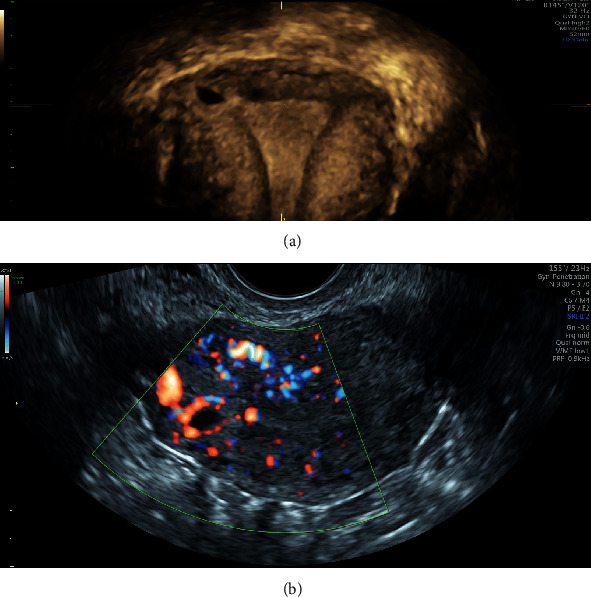
(a) Reconstruction 3D; (b) peripheral vascularization of IP.

A 35-year-old Caucasian pluriparous pregnant woman was referred to our department with an ectopic tubal pregnancy diagnosed by her primary care physician. She was completely asymptomatic, with no uterine bleeding or pelvic pain, and no history of previous miscarriages. She was 6 + 6 weeks pregnant based on her last period. An experienced operator performed a TVUS, which revealed a GS without embryo in the interstitial portion of the right tuba, and also detected the interstitial line between the GS and the lateral edge of the endometrial cavity, and the myometrial mantle around the ectopic sac (Figure 2). The patient's serum *β*-hCG was 2664 mUi/mL. We diagnosed an IP and it started a treatment with 600 mg of oral mifepristone with a single systemic dose of MTX, consisting of an intramuscular injection (IM) of 50 mg/m2 of body surface area according to Stovall et al. protocol, being the patient hemodynamically stable asymptomatic, and at an early gestational age. Serum *β*-hCG was checked on day 0^th^, on day 4^th^ (2952 mUI/mL), and 7^th^ (1772 mUI/mL) after treatment, with a constant lowering of *β*-hCG levels. She also underwent a sonographic evaluation on days 0^th^, 4^th^, and 7^th^, and 14 days after the medical treatment, and *β*-hCG levels continued to decrease (992 mUI/mL). Unexpectedly, after 21 days, there was an increase of the serum *β*-hCG level (1117 mUI/mL) without symptoms or modification in ultrasound images. We decided to administrate a second injection of MTX. Ultrasound examinations were performed weekly until a complete resolution of the IP. Subsequently, on day 28^th^ from the first dose was recorded a reduction of the *β*-hCG to 694.6 mUI/L, and we achieved complete negativization of Serum *β*-hCG in 47 days. (Table 1).

## 3. Results and Discussion

The improving efficacy of medical treatment in the case of IP requires an early diagnosis, and advances in TVUS and availability of quantitative *β*-hCG have made it possible.

IP could be asymptomatic until 7-16 gestational weeks [3]. There is no evidence of a specific serum *β*-hCG trend that is sensitive in differentiating IP from either healthy intrauterine pregnancies or other types of EP. The TVUS examination is essential for the early and differential diagnosis of the IP from isthmic pregnancy, particularly in stump pregnancy. The eccentric position of the GS and the thinning of the myometrial mantle make the differential diagnosis between eccentric (angular/cornual) and interstitial pregnancies difficult. Timor-Tritsch outlined three US criteria for IP diagnosis [4]: an empty uterine cavity, a myometrial layer of less than 5 mm surrounding the GS, and a chorionic sac separated and laterally located 1 cm or more from the sideward portion of the uterine cavity. Jurkovic and Mavrelos have proposed a combination of two diagnostic outcomes for interstitial pregnancy: visualization of the interstitial line between the gestational sac and the lateral edge of the endometrial cavity and the myometrial mantle around the ectopic sac. 3-D ultrasound facilitates the visualization of the interstitial portion of the tube and can be useful in differentiating intrauterine from interstitial pregnancies [5]. According to a recent review of Ackerman et al., the diagnostic accuracy could increase with the interstitial line sign, which is a US mark that reaches 80% sensitivity and 98% specificity [6]. In the case of inconclusive TVUS examination, it is possible to diagnose IP by RMI in clinically stable patients, or by a laparotomic or laparoscopic approach in hemodynamically unstable patients. Alternative treatments need to be tailored on a case by case and are related to the gestational age at the time of diagnosis, clinical presentation, and desire for future pregnancies. Expectant management could be a first-line approach in selected asymptomatic patients with a spontaneous miscarriage or at an early presentation [7]. The main drawbacks include uterine rupture with a substantial increase in maternal morbidity/mortality, the need for prolonged hospitalization, and the risk of recurrence [1, 3, 8, 9]. It is an appropriate first-line approach for women with an IP and declining serum *β*-hCG levels (regardless of ectopic mass size and initial serum *β*-hCG levels).

Conservative management could be a viable option for most of the cases of early IP and it is related to the basal serum *β*-hCG [5–10]. The medical therapy involves the use of MTX, injected locally, close or into the GS (under TVUS or laparoscopic guidance), or systemic, with a success rate that depends on the administration route, single, or multidose regimen [3]. MTX has a well-known role in selected ectopic pregnancy since 1982 [11], and a recent review highlighted its reliable application regarding uncomplicated IP [3]. The dose of one-off systemic MTX is calculated as 50 mg/m2 body surface area, checking of the serum *β*-hCG level, according to the Stovall protocol, after the treatment, on the same day, on the 4th, and 7th. When *β*-hCG levels are lower than 5000, a single dose of systemic MTX should be sufficient [12].

Although the single-dose protocol has been reported as consistent treatment [13], according to Barnhart et al., the multidose regimen is more effective [14]. Also in our second case, the single-dose protocol resulted inadequate despite of a low serum *β*-hCG. In patients with a continuous rise of *β*-hCG, sonographic signs of pregnancy progression (e.g., the development of fetal cardiac activity), we should consider repeating the administration of MTX [15]. Conti et al. recently obtained a complete resolution of interstitial pregnancy with *β*-hCG levels of 35,993 mUI/mL. They treated the patients with multiple dosing of MTX plus folinic acid for 5 days obtaining the complete negativization of *β*-hCG levels after more than one month (Table 1) [16]. However, the multiple-dose of MTX has potentially dangerous side effects, such as bone marrow suppression and granulocytopenia [12], and there is not a consensus on which therapy may be the most effective in treating IPs.

Recent studies have reported that a pharmacological approach using MTX is usually effective, although there is insufficient evidence to recommend a local or systemic approach [13]. Local administration of MTX, either transvaginal or laparoscopic, can be safer than systemic, with a lower incidence of side-effects, smaller dosage, and higher tissue concentration; however, it is more invasive and requires special facilities and trained personnel. In cases of heterotopic pregnancy, the administration of MTX is possible only if the intrauterine pregnancy is nonviable or if the woman does not wish to continue with the pregnancy (level of evidence D). It is possible performing a TVUS-guided aspiration of the extracelomic fluid from the gestational sac, followed by intrasaccular injection of 25 mg of MTX with/without 0.2–0.4 mEq of potassium chloride in clinically stable patients [9].

Probably, another factor that could influence the choice of the therapy (local or systemic) may be the vascularization of IP during the US evaluation. A higher vascularization of the peripheral portion of IP could lead to a better outcome of the therapy with systemic MTX considering the possibility of bleeding during invasive procedures for local injection and the capacity of the drug to reach the target in case of large vascularization.

Brincat et al., in his recent review, reported any significant difference in the success rate between systemic and local MTX (success rate for systemic MTX was 79.9% (95% CI 72.68–87.29); success rate for local MTX injection 97.83% (95% CI 93.59–100) [15].

None of the studies reported any statistical significant difference between two types of treatment (Table 1).

The combination of mifepristone with MTX to treat ectopic pregnancy was first reported by Perdu et al. in 1998 in a descriptive study that included a total of 30 patients diagnosed with ectopic tubal pregnancy with *β*-hCG levels <10,000 mIU/mL with a low failure rate estimated at 3.3% [17]. This research highlighted the possible synergy between the two drugs that can induce the trophoblast cell lysis more rapidly than the MTX monotherapy. Mifepristone is a steroidal antiprogestogen drug that can competitively combine with progesterone receptor and glucocorticoid receptor, inhibit the activity of progesterone, and lead to cell degeneration and the decrease of decidua and chorion. Moreover, mifepristone promotes the release of endogenous prostaglandin, which will trigger uterine contraction, cervix softening, and dilation to assist in ectopic embryonic tissues discharging [18]. Probably his effect on decidua is the reason of his efficacy in the treatment of intrauterine pregnancy or interstitial pregnancy. While in tubal pregnancy, its effect is reduced by the presence of a discontinuous deciduous islet. Rozenberg et al. made the first randomized, double-blind, placebo-controlled trial, comparing the efficacy of MTX ± mifepristone versus MTX ± placebo in the medical management of the ectopic pregnancy, and demonstrated that the adjunct of mifepristone did not increase the efficacy of MTX instead, when progesterone level was >10 ng/L, the efficacy of the combination of mifepristone and MTX resulted significantly higher (83.3% success rate (15/18) versus 38.5% (5/13)) [19]. In this study, when the serum *β*-hCG level was <1500 mIU/mL, the success rate of MTX plus mifepristone was 90.6%, probably due to a luteolytic effect of mifepristone even in the case of a very active corpus luteum. Recently, Gomez et al. reported the successful management of four cases (two IP and two cervical pregnancies), treated with a single dose of IM-MTX combined with oral Mifepristone 600 mg. These two IP had a baseline *β*-hCG of less than 5,000 mIU/mL, with a blighted ovum and an embryo with no cardiac activity [20]. Another case was reported in the literature about the possibility of managing IP in an early stage and in presence of *β*-hCG levels less than 5,000 mIU/mL with a single dose of Mifepristone 200 mg along with an injection of MTX 50 mg intramuscularly, thus obviating the need for a surgical intervention (Table 1) [21].

In our first case, we successfully treated the patient with the association Mifepristone 600 mg orally and injection of 1 mg/kg IM of MTX, plus a single dose of 0.1 mg folinic acid, even in the presence of an embryo with cardiac activity and high serum *β*-hCG levels (19397 mUi/mL). We assume that this new therapeutic scheme could be considered as a valid option for asymptomatic women with IPs for the synergic role of MTX and Mifepristone in contrasting GS implantation, also in the presence of a vital embryo.

Otherwise, in our second case, in presence of serum *β*-hCG levels <5000 mUi/mL (2664 mUi/mL) and with no evidence of embryo echoes, we decided to treat the patient with the association of Mifepristone 600 mg and MTX 50 mg/m2 of body surface IM; a second dose of systemic MTX had to be administrated after 21 days because of persisting high *β*-hCG levels. According to the present literature, we retain that the single-dose protocol is associated with a higher rate of failure.

Probably the evaluation of pregnancy vascularization could be diriment for the choice of combined therapy. A higher vascularization could indicate the presence of a wider syncytiotrophoblast with its progesterone secretion, therefore, less susceptible to therapy with the Mifepristone.

There is no unanimity also on the best surgical procedure for interstitial ectopic pregnancy. The evolution of minimally invasive surgery has provided us with more therapeutic options for the treatment of ectopic pregnancies [16, 22]. The surgical laparotomy treatment is the only appropriate route in case of unstable hemodynamic women with a rupture suspicion or recurrent IP [3]. More conservative surgical approaches have been proposed, and currently, laparoscopy is the most adopted technique in elective surgery [3]. Cornual or minicornual resection could be addressed to viable IP with a previous history of failed therapeutic strategy [23], instead of a cornuostomy that could be adopted with an IP of less than 4 cm in diameter [24]. Last year, Pramayadi et al. successfully treated a 35-year-old woman with cornual pregnancy using laparoscopic cornuostomy. However, the same authors in the discussion of their study affirmed that the laparoscopy approach needs a high-skilled laparoscopic surgeon. Moreover, they used vasopressin to minimalize bleeding, assuming the risks linked to this drug, such as cardiovascular adverse effects, if systemic injection occurs (severe hypertension, myocardial infarction, and acute pulmonary edema) [25]. In selected cases, including hemodynamic stability and no evidence of uterine rupture, it is possible to use new minimally invasive techniques: laparoscopic-guided transcervical evacuation, laparoscopic-guided use of the resectoscope, ipsilateral uterine artery ligation at the time of the cornual repair, and the use of end loop and encircling sutures at the cornua. Other authors in 2009 had described a novel approach for the treatment of IP by using laparoscopic salpingocentesis, MTX local injection (50 mg/m2) after aspiration of the amniotic fluid, the remainder dose was given intramuscularly, and oral Mifepristone 200 mg that was administered postoperatively [26].

In literature was described the successful treatment of IPs also by selective uterine artery embolization without any severe complications. Blocking the blood flow in the uterine artery may decrease the vascularization of the pregnancy in the interstitial area, with the subsequent trophoblastic degeneration. The risk of uterine rupture during subsequent pregnancies in patients who have been previously treated for an interstitial pregnancy has not been clearly established [27].

Ruptured uterus during subsequent pregnancy has been described after spontaneous resolution or surgical treatment of IP. Angular pregnancy can progress to the second trimester or even to term but is associated with high rates of spontaneous abortion, uterine rupture, and placenta accreta, with rupture occurring in as many as 23.5% of all angular pregnancies [28, 29].

Medical counseling is required before a subsequent conception.

## 4. Conclusions

Clinical management of IP remains a debated topic, and there is no consensus or guidelines for choosing one treatment over another.

According to the present literature, our paper is the first to deal with a combination of MTX multidose and oral mifepristone to treat IP with *β* − hCG level > 10.000 UI/mL and with vital embryo.

The treatment should be personalized considering the obstetric history of the patients, the gestational age at the diagnosis, and their desire for future pregnancies. In selected cases, we proposed a multidose MTX IM regimen combined with mifepristone (600 mg orally administered) in asymptomatic women with low serum *β*-hCG levels at an early gestational age and it can be considered also in asymptomatic women with strong motivation for future conceptions, although in case of high serum *β*-hCG.

Further studies using prospective data from multiple centers are required to establish which is the best approach for IP management.

## Figures and Tables

**Table 1:** Management of IPs with MTX and Oral Mifepristone: *β*-hCG: Beta Human Chorionic Gonadotropin; MTX: Methotrexate; CRL: crown-rump length; IM: intramuscular.

Reference	No. cases	Initial *β*-hCG (mUi/mL)	Management	Time until *β*-hCG undetectable (days)	Outcomes
Case1	1	19397	Mifepristone 600 mg + MTX 1 mg/kg + 0.1 mg Folinic acid	30	Complete resolution (presence of an embryo with CRL 3.6 mm)
Case2	1	2664	Mifepristone 600 mg + MTX 50 mg/m^2^ of body surface (two doses)	47	Complete resolution (no evidence of embryo)
Bremner et al. 2000 [24]	2	3724-4116	Oral mifepristone 600 mg + IM MTX 50 mg/m^2^ of body surface	35-49	Complete resolution
Karki and Saha 2016 [21]	1	594,8	Oral mifepristone 200 mg + IM MTX 50 mg/m^2^ of body surface	42	Complete resolution
Narang at Kalu 2009 [26]	2	8465-3700	Laparoscopic salpingocentesis+local MTX 50 mg/m^2^ + IM MTX 50 mg/m^2^ of body surface+oral mifepristone 200 mg	—	Complete resolution

## Data Availability

All data generated or analysed during this study are included in this published article and its supplementary information files.

## Abbreviations

IP: Interstitial pregnancy
EP: Ectopic pregnancy
*β*-hCG: Beta-human chorionic gonadotropin
MTX: Methotrexate
TVUS: Transvaginal sonography
GS: Gestational sac
CRL: Crown-rump length
IM: Intramuscular
US: Ultrasound.

## Supplementary Materials

Supplementary Materials “Guidelines for the manuscript. Care Checklist”.

## References

[1] Jermy K., Thomas J., Doo A., Bourne T. (2004). The conservative management of interstitial pregnancy. *BJOG: An International Journal of Obstetrics & Gynaecology*.

[2] Chen J., Huang D., Shi L., Zhang S. (2019). Prevention, diagnosis, and management of interstitial pregnancy: a review of the literature. *Laparoscopic, Endoscopic and Robotic Surgery*.

[3] Moawad N. S., Mahajan S. T., Moniz M. H., Taylor S. E., Hurd W. W. (2010). Current diagnosis and treatment of interstitial pregnancy. *American Journal of Obstetrics and Gynecology*.

[4] Timor-Tritsch I. E., Monteagudo A., Matera C., Veit C. R. (1992). Sonographic evolution of cornual pregnancies treated without surgery. *Obstetrics and Gynecology*.

[5] Hafner T., Aslam N., Ross J. A., Zosmer N., Jurkovic D. (1999). The effectiveness of non-surgical management of early interstitial pregnancy: a report of ten cases and review of the literature. *Ultrasound in Obstetrics & Gynecology*.

[6] Ackerman T. E., Levi C. S., Dashefsky S. M., Holt S. C., Lindsay D. J. (1993). Interstitial line: sonographic finding in interstitial (cornual) ectopic pregnancy. *Radiology*.

[7] Stovall T. G., Ling F. W. (1993). Single-dose methotrexate: an expanded clinical trial. *American Journal of Obstetrics and Gynecology*.

[8] Liao C.-Y. (2018). Distinguishing between interstitial and angular pregnancies: Is there a role for saline infusion sonohysterography?. *Taiwanese Journal of Obstetrics and Gynecology*.

[9] Di Tizio L., Spina M. R., Gustapane S., D’Antonio F., Liberati M. (2018). Interstitial pregnancy: from medical to surgical approach—report of three cases. *Case Reports in Obstetrics and Gynecology*.

[10] Chetty M., Elson J. (2009). Treating non-tubal ectopic pregnancy. *Best Practice & Research Clinical Obstetrics & Gynaecology*.

[11] Tanaka T., Hayashi H., Kutsuzawa T., Fujimoto S., Ichinoe K. (1982). Treatment of interstitial ectopic pregnancy with methotrexate: report of a successful case. *Fertility and Sterility*.

[12] Conti V., Luciano G., Pecoraro G., Iovieno R., Filippelli A., Guida M. (2018). Multidosing intramuscular administration of methotrexate in interstitial pregnancy with very high levels of *β*-hCG: a case report and review of the literature. *Frontiers in Endocrinology*.

[13] Zalel Y., Caspi B., Insler V. (1994). Expectant management of interstitial pregnancy. *Ultrasound in Obstetrics & Gynecology*.

[14] Barnhart K. T., Gosman G., Ashby R., Sammel M. (2003). The medical management of ectopic pregnancy: a meta-analysis comparing “Single dose” and “multidose” regimens. *Obstetrics and Gynecology*.

[15] Brincat M., Bryant-Smith A., Holland T. K. (2019). The diagnosis and management of interstitial ectopic pregnancies: a review. *Gynecological Surgery*.

[16] Mangino F. P., Romano F., Di Lorenzo G. (2019). Total hysteroscopic treatment of cervical pregnancy: the 2-step technique. *Journal of Minimally Invasive Gynecology*.

[17] Perdu M., Camus E., Rozenberg P. (1998). Treating ectopic pregnancy with the combination of mifepristone and methotrexate: a phase II nonrandomized study. *American Journal of Obstetrics and Gynecology*.

[18] Wan S., Xiang Y., Fang W., Huang D. (2016). The effect of methotrexate in combination with mifepristone on ectopic pregnancy: a meta-analysis. *International Journal of Clinical & Experimental Medicine*.

[19] Garbin O., De Tayrac R., De Poncheville L. (2004). Medical treatment of ectopic pregnancy: a randomized clinical trial comparing metotrexate-mifepristone and methotrexate-placebo. *Journal de Gynecologie, Obstetrique et Biologie de la Reproduction*.

[20] Gómez García M. T., Aguarón Benitez G., Barberá Belda B., Callejón Rodríguez C., González Merlo G. (2012). Medical therapy (methotrexate and mifepristone) alone or in combination with another type of therapy for the management of cervical or interstitial ectopic pregnancy. *European Journal of Obstetrics, Gynecology, and Reproductive Biology*.

[21] Karki U., Saha R. (2016). Medical management of unruptured interstitial pregnancy with mifepristone and methotrexate. *Journal of Kathmandu Medical College*.

[22] Tantchev L., Kotzev A., Yordanov A. A. (2019). Disturbed interstitial pregnancy: a first case of successful treatment using a mini-laparoscopic approach. *Medicina*.

[23] Moawad N. S., Dayaratna S., Mahajan S. T. (2009). Mini-cornual excision: a simple stepwise laparoscopic technique for the treatment of cornual pregnancy. *JSLS: Journal of the Society of Laparoendoscopic Surgeons*.

[24] Bremner T., Cela V., Luciano A. A. (2000). Surgical management of interstitial pregnancy. *The Journal of the American Association of Gynecologic Laparoscopists*.

[25] Pramayadi C. T., Bramantyo A., Gunardi E. R. (2018). Successful procedure in conservative management of interstitial (cornual) ectopic pregnancy. *Gynecology and Minimally Invasive Therapy*.

[26] Narang L., Kalu G. (2009). Laparoscopic salpingocentesis using methotrexate in combination with oral mifepristone for successful treatment of interstitial pregnancy: a case report. *Fertility and Sterility*.

[27] Grindler N. M., Ng J., Tocce K., Alvero R. (2016). Considerations for management of interstitial ectopic pregnancies: two case reports. *Journal of Medical Case Reports*.

[28] Yassin A. S., Taha M. S. (2017). Interstitial ectopic pregnancy, Diagnosis and management: a case report and literature review. *Annals of Clinical Case Reports*.

[29] Faraj R., Steel M. (2007). Management of cornual (interstitial) pregnancy. *The Obstetrician & Gynaecologist*.

