# Supplementary material for: Conservative Treatment of Interstitial Ectopic Pregnancy with the Combination of Mifepristone and Methotrexate: Our Experience and Review of the Literature
Source: Biomed Res Int. 2020 Aug 1;2020:8703496. doi: 10.1155/2020/8703496 (PMC7421079; doi:10.1155/2020/8703496)
Supplement: Supplementary Materials — “Guidelines for the manuscript. Care Checklist”. [file 8703496.f1.pdf]

CARE
case report guidelines

CARE Checklist of information to include when writing a case report

(cc) BY-NC-ND

Topic	Item	Checklist item description	Reported on Line
Title	1	The diagnosis or intervention of primary focus followed by the words "case report"	lines 1-2 ; pag. 1
Key Words	2	2 to 5 key words that identify diagnoses or interventions in this case report, including "case report"	line 31; pag. 2
Abstract (no references)	3a	Introduction: What is unique about this case and what does it add to the scientific literature?	lines 14-15; pag. 1
	3b	Main symptoms and/or important clinical findings	lines 17-19 pag.1
	3c	The main diagnoses, therapeutic interventions, and outcomes	lines from 19 to 22; pag. 1
	3d	Conclusion—What is the main "take-away" lesson(s) from this case?	lines 26-27; pag.1
Introduction	4	One or two paragraphs summarizing why this case is unique (**may include references**)	lines from 40 to 46; pag.2
Patient Information	5a	De-identified patient specific information.	CASE1 lines 51 ;pag 3 /CASE2 line 66 pag.3
	5b	Primary concerns and symptoms of the patient.	CASE1 lines from 52 to 57; pag.3 /CASE2 lines 67-71 ; pag.3
	5c	Medical, family, and psycho-social history including relevant genetic information	CASE1 lines 51-52 pag.3 /CASE2 lines 66 ; pag.3
	5d	Relevant past interventions with outcomes	CASE1 lines 51-52 pag.3 /CASE2 lines 66 ; pag.3
Clinical Findings	6	Describe significant physical examination (PE) and important clinical findings.	CASE1 lines from 51 to 57; pag.3 /CASE2 lines 67to 71; pag.3
Timeline	7	Historical and current information from this episode of care organized as a timeline	N/A
Diagnostic Assessment	8a	Diagnostic testing (such as PE, laboratory testing, imaging, surveys).	CASE1 lines from 51 to 57; pag.3 /CASE2 lines 67to 71; pag.3
	8b	Diagnostic challenges (such as access to testing, financial, or cultural)	N/A
	8c	Diagnosis (including other diagnoses considered)	CASE1 line 54-56; pag. 3 / CASE2 line 68 to 71 ; pag 3
	8d	Prognosis (such as staging in oncology) where applicable	N/A
Therapeutic Intervention	9a	Types of therapeutic intervention (such as pharmacologic, surgical, preventive, self-care)	CASE1 lines 59 to 62 pag.3/ CASE2 lines 72-78; pag.3
	9b	Administration of therapeutic intervention (such as dosage, strength, duration)	CASE1 lines 59 to 62 pag.3/ CASE2 lines 72-78; pag.3
	9c	Changes in therapeutic intervention (with rationale)	N/A
Follow-up and Outcomes	10a	Clinician and patient-assessed outcomes (if available)	CASE1 lines 64-65 pag.3/ CASE2 lines 79 to 81; pag.3
	10b	Important follow-up diagnostic and other test results	CASE1 lines from 61 to 65 pag 3/ CASE2 lines from 74 to 81 pag 3-4
	10c	Intervention adherence and tolerability (How was this assessed?)	N/A
	10d	Adverse and unanticipated events	N/A
Discussion	11a	A scientific discussion of the strengths AND limitations associated with this case report	N/A
	11b	Discussion of the relevant medical literature **with references**.	lines from 98 to 153; pag. 4 to 6; lines from 166 to 186 pag 7
	11c	The scientific rationale for any conclusions (including assessment of possible causes)	lines from 153 to 165; pag. 6
	11d	The primary "take-away" lessons of this case report (without references) in a one paragraph conclusion	lines from 188 to 198; pag. 7-8
Patient Perspective	12	The patient should share their perspective in one to two paragraphs on the treatment(s) they received	N/A
Informed Consent	13	Did the patient give informed consent? Please provide if requested	Yes ☑ No ☐
